# COVID-19 Knowledge, Attitudes, and Practices Among People in Bangladesh: Telephone-Based Cross-sectional Survey

**DOI:** 10.2196/28344

**Published:** 2021-11-05

**Authors:** Md Golam Rabbani, Orin Akter, Md Zahid Hasan, Nandeeta Samad, Shehrin Shaila Mahmood, Taufique Joarder

**Affiliations:** 1 Public Health Foundation, Bangladesh Dhaka Bangladesh; 2 Health Systems and Population Studies Division International Centre for Diarrhoeal Disease Research, Bangladesh Dhaka Bangladesh; 3 Department of Public Health North South University Dhaka Bangladesh

**Keywords:** COVID-19, knowledge, attitude, practice, risk communication and community engagement, social and behavior change communication, Bangladesh, COVID-19, risk, pandemic, risk communication

## Abstract

**Background:**

The world has been grappling with the COVID-19 pandemic, a dire public health crisis, since December 2019. Preventive and control measures have been adopted to reduce the spread of COVID-19. To date, the public’s knowledge, attitudes, and practices regarding COVID-19 across Bangladesh have been poorly understood. Therefore, it is important to assess people’s knowledge, attitudes, and practices (KAP) toward the disease and suggest appropriate strategies to combat COVID-19 effectively.

**Objective:**

This study aimed to assess the KAP of Bangladeshi people toward COVID-19 and to identify their determinants.

**Methods:**

We conducted a country-wide cross-sectional telephonic survey from May 7 to 29, 2020. A purposive sampling method was applied, and adult Bangladeshi citizens who have mobile phones were approached to participate in the survey. Interviews were conducted based on verbal consent. Multiple logistic regression analyses and several tests were performed to identify the factors associated with KAP related to COVID-19.

**Results:**

A total of 492 of 576 Bangladeshi adults aged 18 years and above completed the interview, with a response rate of 85.4% (492/576). Of the 492 participants, 321 (65.2%) were male, and 304 (61.8%) lived in a rural area. Mean scores for knowledge, attitudes, and practices were 10.56 (SD 2.86), 1.24 (SD 0.83), and 3.17 (SD 1.5), respectively. Among the 492 respondents, 273 (55.5%) had poor knowledge, and 251 (49%) expressed a negative attitude; 192 out of 359 respondents (53.5%) had poor practices toward COVID-19. Mean scores of knowledge, attitudes, and practices differed significantly across various demographic and socioeconomic groups. Rural residents had lower mean scores of knowledge (mean 9.8, SD 3.1, *P*<.001) and adherence to appropriate practice measures (mean 4, SD 1.4, *P*<.001) compared to their urban counterparts. Positive and statistically strong correlations between knowledge and attitudes (*r*=0.21, *P*<.001), knowledge and practices (*r*=0.45, *P*<.001), and attitudes and practices (*r*=0.27, *P*<.001) were observed. Television (53.7%) was identified as the major source of knowledge regarding COVID-19. Almost three-quarters of the respondents (359/492, 73%) went outside the home during the lockdown period. Furthermore, the study found that good knowledge (odds ratio [OR] 3.13, 95% CI 2.03-4.83, and adjusted OR 2.33, 95% CI 1.16-4.68) and a positive attitude (OR 2.43, 95% CI 1.59-3.72, and adjusted OR 3.87, 95% CI 1.95-7.68) are significantly associated with better practice of COVID-19 health measures.

**Conclusions:**

Evidence-informed and context-specific risk communication and community engagement, and a social and behavior change communication strategy against COVID-19 should be developed in Bangladesh based on the findings of this study, targeting different socioeconomic groups.

## Introduction

Since the end of 2019, the world has been confronting the continuous morbidity and mortality caused by COVID-19 [1,2]. Bangladesh is one of the most affected countries, and it is currently experiencing a new surge of infections. As of March 25, 2021, there had been reports of over 125 million confirmed cases of COVID-19, including more than 2.75 million deaths globally [3]. As of March 22, 2021, Bangladesh was among the top 34 countries, with 0.47% of the total global COVID-19 cases [4].

In Bangladesh, the first confirmed COVID-19 cases and death were reported on March 8 and 18, 2020, respectively [5]. Since then, the pandemic has spread gradually over the whole country, and the number of affected people has been increasing [4]. It has been presumed that the consequences of COVID-19 will be devastating in Bangladesh as, with a population of approximately 165 million, it is the second most densely populated country in the world, with approximately 1240 people per square kilometer [6-8]. Moreover, a large proportion of the population still lives below the poverty line, and almost half of the population is exposed to multiple socioeconomic vulnerabilities [6,9]. Further, Bangladesh suffers from a low literacy rate [7], potentially exposing the population to unfavorable knowledge, attitudes, and practices (KAP) toward a persisting pandemic. Evidence has shown that socioeconomically vulnerable communities lag in access to health, education, and information, which may aggravate the spread of an infectious disease [10,11].

In a short time, after the identification of the first case, Bangladesh was recognized as the second most affected country in South Asia. To control the spread of COVID-19, therefore, the government declared a nationwide “general holiday” (lockdown) for a few months and recommended that its people maintain the standard guidelines [12-14]. Since the beginning of the pandemic, control of the spread of SARS-CoV-2 has been considered the most effective measure to protect people. Thus, regarding preventing the spread of COVID-19, nonclinical interventions such as restricting unnecessary movement, maintaining social distance, wearing masks, and frequent and appropriate handwashing have been suggested by the World Health Organization (WHO) [15-17]. Maintaining such interventions largely depends on people’s KAP, as existing scientific evidence has shown that sociodemographic and economic characteristics are associated with the level of people’s knowledge and attitudes, which safeguard against disease spread [10,18].

Inadequate knowledge, negative attitudes, and poor practices toward an infectious disease may lead to superfluous threats, rumors, confusion, and even unnecessary fear, which may aggravate the pandemic. Misconception, contradictory perception, and practice of unscientific remedies for COVID-19 were found among Bangladeshi people. Thus, it is important to understand the KAP regarding COVID-19 among the general population to prevent the spread of the infection. This study will contribute to filling the gap that currently exists. The objective of this study was to assess the KAP toward COVID-19 of Bangladeshi residents. The findings from this study are expected to help policy makers design context-specific social and behavior change strategies for the population in Bangladesh and in other similar settings.

## Methods

### Study Design and Setting

A cross-sectional telephone survey was conducted applying a quantitative approach. The survey was conducted from May 7 to 29, 2020, during the lockdown period in Bangladesh. The study was conducted in all 8 administrative divisions (Barishal, Chattogram, Dhaka, Khulna, Mymensingh, Rajshahi, Rangpur, and Sylhet) of the country.

### Recruitment Procedure

**Figure 1:**
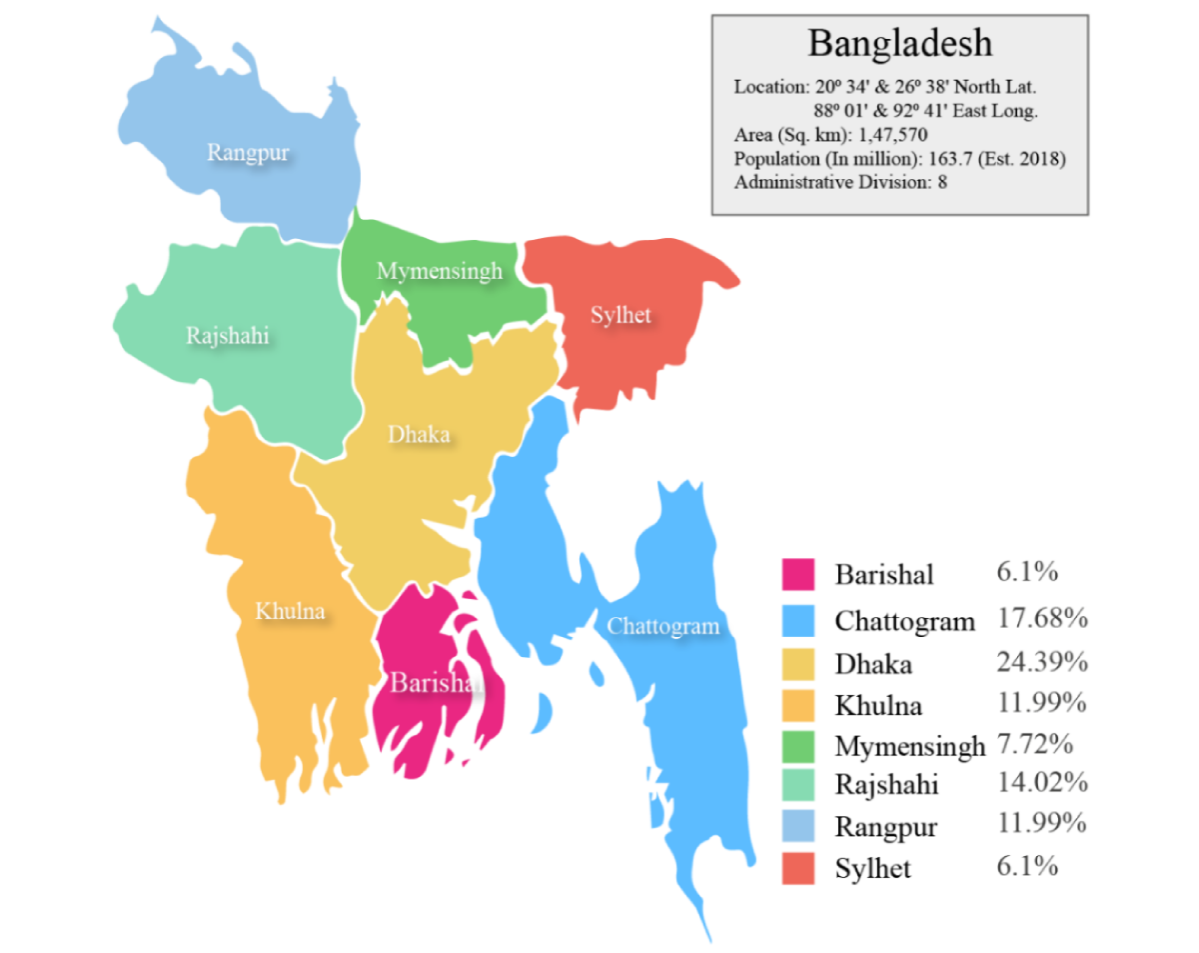
Proportion of respondents who completed interviews in 8 administrative divisions of Bangladesh.

A purposively selected sample of individuals was interviewed to measure the KAP toward COVID-19. The inclusion criteria included Bangladeshi citizens with a minimum age of 18 years who are in possession of a mobile phone. The researchers collected contact numbers of the study population through direct contact or via their network, consisting of family members, friends, and relatives, regardless of their demographic and socioeconomic status according to the inclusion criteria. To ensure the fulfillment of the inclusion criteria, we asked the respondents about their birth year and whether they had a national identity card (which is only available to Bangladeshis over the age of 18 years). When collecting contact numbers, the people were informed that they might be contacted for an interview. With this method, we developed a pool of eligible and initially interested respondents. Before data collection, we estimated the sample size. We assumed that 50% (*P*=.05) of the population has the proper KAP toward COVID-19. With a 95% confidence level and 5% precision level, a representative sample of 384 was estimated using this proportion. The sample size increased to 460 and 576 after adjusting for the 1.2 design effect and the 20% nonresponse rate. We approached 576 individuals who were interested in taking part in this study from 8 divisions proportionately to the population of the respective divisions (Barishal 5.7%, Chattogram 17.5%, Dhaka 23.3%, Khulna 11.9%, Mymensingh 7.4%, Rajshahi 14.3%, Rangpur 11.8%, and Sylhet 6%) [19] (Figure 1), of whom 504 individuals agreed to participate in the interview and another 72 individuals were not interested in participating in the interview. A total of 492 completed interviews were used to analyze data after excluding incomplete interviews.

### Study Instrument and KAP Measure

We developed a questionnaire (Multimedia Appendix 1) by following the national guidelines for clinical and community management of COVID-19 introduced by the Government of Bangladesh, WHO reports, and literature [15,20-22]. The questionnaire was then translated into Bengali, contextualized for Bangladesh, reviewed by 2 sectoral experts, piloted in 15 adult people, and finalized to ensure the respondents understood all the KAP-related questions. The individual survey questionnaire consisted of two sections. The first section focused on sociodemographic and economic characteristics, including age, gender, education, occupation, current residence, religion, marital status, number of household members, living conditions, toilets in the respondent’s current residence, and income level. The second section covered KAP-related questions (including 14 items for knowledge, 2 items for attitudes, and 6 items for practices), and the overall Cronbach α of KAP questions regarding the final data was .86, which indicates a satisfactory level of internal consistency [23].

The knowledge questions focused on clinical presentations of COVID-19, transmission routes, prevention, and infection control. The responses to these questions included “yes” and “no” options, with an additional “don’t know” option. A correct answer was assigned a value of 1, and an incorrect or “don’t know” answer was assigned a value of 0. The total knowledge score ranged from 0 (no correct answers) to 14 (all correct answers), with a higher score indicating better knowledge of COVID-19. To determine the knowledge level, following Bloom’s cutoff point [24], we determined that 80% to 100% positive responses indicated good knowledge and percentages of positive responses below that indicated poor knowledge.

A similar scoring approach was used for classifying positive and negative attitudes and for good and poor practices. To measure attitudes toward COVID-19, selected participants were asked 2 questions: whether they agreed, disagreed, or were not sure that the pandemic would be successfully controlled across the world, and their level of confidence that Bangladesh would win the battle against COVID-19.

Similarly, to measure practices, 5 self-reported statements were used, namely, whether respondents had avoided crowded places while being outside; maintained the recommended social distance of 1 m (or approximately 3 feet); worn a mask regularly and correctly when outside; washed their hands regularly and thoroughly after coming from outside and before eating or touching the mouth, nose, or eyes; and maintained the recommended hand washing duration of 20 to 30 seconds each time. However, for practice data, we only concentrated on the high-risk population in terms of mobility. We purposively collected information on practices toward COVID-19 only from those respondents who left their homes during the COVID-19 lockdown period. The complete set of data collected is available in Multimedia Appendix 2.

### Statistical Analysis

Both descriptive and inferential statistical analyses were performed. In the descriptive analyses, the characteristics of the study participants were presented in terms of frequency and percentage with a 95% confidence interval. The Wilcoxon rank sum test for 2 categorical variables and Kruskal-Wallis test for more than 2 categorical variables were performed to test the statistical inferences across demographic and socioeconomic information and KAP scores, respectively. The Spearman rank correlation test was performed to assess the correlations between the scores of knowledge and attitudes, knowledge and practices, and attitudes and practices. Multiple logistic regression analyses were used to assess the association of the demographic and socioeconomic characteristics of the respondents with their levels of knowledge, attitudes, and practices separately. Furthermore, the associations among knowledge and attitudes, knowledge and practices, and attitudes and practices were assessed to verify the internal consistency. In this analysis, the degree of knowledge, type of attitude, and level of practice were considered dependent variables, whereas age group, sex, education level, occupation, and other variables were considered independent variables. A *P* value <.05 was considered statistically significant. We analyzed the data using Stata SE, version 13.0 (StataCorp LLC).

### Ethics Approval and Consent of Respondents

The research protocol, questionnaire, consent statement, and procedures of obtaining informed consent were approved by the Ethical Review Committee (2020/01) of the Public Health Foundation, Bangladesh. As the interview was conducted over the telephone, informed verbal consent was obtained from all respondents before the interview. Respondents were informed in Bengali about their rights to voluntary participation and to withdraw from the interview at any point. Respondents were assured of the anonymity of the data they provided.

## Results

### Demographic and Socioeconomic Characteristics

A total of 492 individuals were interviewed in this study, and the majority of them belonged to a younger age group (n=256, 52%, aged ≤35 years). Among the 492 respondents, 321 (65.2%) were male, 156 (31.7%) had a bachelor’s degree or higher level of education, 304 (61.8%) were from a rural area, 202 (41%) were unemployed and had no income, and 449 (91.3%) had access to available running water. Other characteristics are shown in Table 1. Moreover, Multimedia Appendix 3 presents the demographic and socioeconomic characteristics of the respondents by administrative division.

**Table 1:** Demographic and socioeconomic characteristics of the respondents (N=492).

Characteristic		Values	
		n (%)	95% CI
**Age group (years)**			
	≤25	84 (17.1)	14-20.7
	26-35	172 (35)	30.9-39.3
	36-45	83 (16.9)	13.8-20.5
	46-55	59 (12)	9.4-15.2
	56-65	54 (11)	8.5-14.1
	≥66	40 (8.1)	6-10.9
**Sex**			
	Male	321 (65.2)	60.9-69.3
	Female	171 (34.8)	30.7-39.1
**Education level**			
	No education	76 (15.5)	12.5-18.9
	Primary	87 (17.7)	14.5-21.3
	Secondary	103 (20.9)	17.6-24.8
	Higher secondary	70 (14.2)	11.4-17.6
	Bachelor’s degree and above	156 (31.7)	27.7-36
**Occupation**			
	Currently unemployed	202 (41.1)	36.8-45.5
	Service holder	134 (27.2)	23.5-31.4
	Farmer	28 (5.7)	4-8.1
	Businessperson	57 (11.6)	9-14.7
	Day laborer	52 (10.6)	8.1-13.6
	Other	19 (3.9)	2.5-6
**Religion**			
	Muslim	454 (92.3)	89.6-94.3
	Other	38 (7.7)	5.7-10.4
**Current residence**			
	Urban	188 (38.2)	34-42.6
	Rural	304 (61.8)	57.4-66
**Marital status**			
	In a marital relationship	331 (67.3)	63-71.3
	Not in a marital relationship	161 (32.7)	28.7-37
**Family size**			
	1-3 members	109 (22.2)	18.7-26.1
	3-6 members	316 (64.2)	59.9-68.4
	>7 members	67 (13.6)	10.9-17
**Earning person in the family**			
	None	6 (1.2)	0.5-2.7
	1 person	253 (51.4)	47-55.8
	≥2 persons	233 (47.4)	43-51.8
**Monthly income (BDT^a^)**			
	No income	202 (41.1)	36.8-45.5
	≤10,000	111 (22.6)	19.1-26.5
	10,001-20,000	80 (16.3)	13.2-19.8
	20,001-3,0000	39 (7.9)	5.8-10.7
	30,001-40,000	33 (6.7)	4.8-9.3
	>40,000	27 (5.5)	3.8-7.9
**Availability of running water at home**			
	Yes	449 (91.3)	88.4-93.5
	No	43 (8.7)	6.5-11.6
**Division of residence**			
	Barishal	30 (6.1)	4.3-8.6
	Chattogram	87 (17.7)	14.5-21.3
	Dhaka	120 (24.4)	20.8-28.4
	Khulna	59 (12)	9.4-15.2
	Mymensingh	38 (7.7)	5.7-10.4
	Rajshahi	69 (14.0)	11.2-17.4
	Rangpur	59 (12)	9.4-15.2
	Sylhet	30 (6.1)	4.3-8.6

^a^BDT: Bangladeshi Taka. 1 BDT=US $.012.

### Knowledge About COVID-19

The mean score of knowledge was estimated at 10.56 (SD 2.86), where the range of scores varied between 0 and 14. The correct response rates regarding the individual knowledge questions ranged between 55.3% (272/492) and 91.5% (450/492). Most of the 492 respondents provided correct responses to the items related to common symptoms (n=447, 90.9%), mode of spreading (n=404, 82.1%), and knowledge about preventive practices, eg, avoiding crowded places (n=409, 83.1%), maintaining recommended social distancing (n=420, 85.4%), and frequent hand washing (n=450, 91.5%) (a detailed distribution is available in Multimedia Appendix 4). Overall, 219 of 492 participants (44.5%) were able to provide correct answers for 11 or more questions out of the total 14 questions, for which they obtained a total score of 11.1 (representing a minimum of 80% of the total score). A score of 11.1 or more was considered to indicate good knowledge about COVID-19 (Table 2).

**Table 2:** Data related to the survey questions, scores, and levels of knowledge, attitudes, and practices regarding COVID-19 (N=492).

Variable	Number of questions	Score		Level, n (%)	
		Range	Total, mean (SD)	Poor or negative	Good or positive
Knowledge	14	0-14	10.56 (2.86)	273 (55.5)	219 (44.5)
Attitudes	2	0-2	1.24 (0.83)	251 (51)	241 (49)
Practices	5	0-5	3.17 (1.50)	192 (53.5)	167 (46.5)

### Attitudes Toward COVID-19

The mean score of attitudes based on the 2 questions in the attitude domain was estimated at 1.24 (SD 0.83, range 0-2). The minimum percentage of respondents providing a positive response for any single question in the attitude domain was 55.3% (272/492), and the maximum percentage of respondents providing a positive response for any single question was 68.5% (337/492). A total of 241 (49%) of the 492 respondents had a strong positive attitude toward the successful control of the pandemic, considering the 2 questions added in the domain. The maximum possible score was 2, which was 0.76 higher than the mean score in the attitude domain (Table 2).

### Practices Around COVID-19

The mean score of practices was estimated at 3.17 (SD 1.50, range 0-5). The minimum percentage of respondents who provided positive answers for any single question in the practices domain was 42.6% (153/492), and the maximum percentage of respondents providing positive answers for any single question was 76% (273/492) (a detailed distribution is available in Multimedia Appendix 2). Respondents scoring between 80% and 100% (equivalent to a score of 4 out of 5) were considered to have good practices toward COVID-19. According to this cutoff, 167 of the 359 respondents (46.5%) who left their homes during the lockdown were found to demonstrate good practices toward COVID-19 (Table 2).

Table 3 shows a comparison of the KAP scores across the respondents’ characteristics. A statistically significant knowledge difference was found between age groups, sexes, education levels, occupations, types of residence, marital relationships, and income quantiles, as well as across divisions. Regarding attitudes, statistically significant variations in attitude scores were found between the religious groups, marital relationships, and administrative divisions. Regarding practices, statistical differences were found between age groups, sexes, levels of education, occupations, religions, types of residence, income quantiles, and water availability at home, as well as across administrative divisions. Similar trends were observed in knowledge and practices: both were comparatively low among the older groups; both knowledge (mean 12.2, SD 1.4; *P*<.001) and practices (mean 5, SD 0.9; *P*<.001) were strongly and positively correlated with a higher level of education. Among different types of professionals, those in the service sector were found to have higher knowledge (mean 12, SD 1.6) and to adopt better practices. On average, rural respondents had significantly lower knowledge (mean 9.8, SD 3.1; *P*<.001) and practice (mean 4, SD 1.4; *P*<.001) scores compared to urban respondents. Regarding the administrative divisions, our results demonstrate that knowledge (mean 7.7, SD 3.6; *P*<.001), attitude (mean 0.8, SD 0.8; *P*<.001), and practice (mean 3.6, SD 1.2; *P*<.001) scores were comparatively low in the Rajshahi division.

**Table 3:** Mean scores of knowledge, attitude, and practice toward COVID-19 among the respondents.

Characteristic			n		Knowledge score, mean (SD)		*P* value			Attitude score, mean (SD)			*P* value			n			Practice score, mean (SD)		*P* value	
**Age** **group^a^ (years)**								<.001						.49								<.001
	≤25	84		11.3 (2)					1.3 (0.8)						58			4.1 (1.1)				
	26-35	172		11.7 (2.1)					1.3 (0.8)						134			4.7 (1.2)				
	36-45	83		10.5 (2.6)					1.3 (0.8)						69			4.2 (1.3)				
	46-55	59		9.2 (3)					1.1 (0.9)						46			4 (1.5)				
	56-65	54		9.1 (3.4)					1.1 (0.8)						34			4.1 (1.3)				
	>66	40		8.1 (3.6)					1.3 (0.8)						18			3.4 (1.6)				
**Sex^b^**								.02						.39								.02
	Male	321		10.8 (2.8)					1.3 (0.8)						266			4.5 (1.2)				
	Female	171		10.2 (3)					1.2 (0.8)						93			3.9 (1.4)				
**Education** **level^a^**								<.001						.65								<.001
	No education	76		8.4 (3.3)					1.3 (0.8)						45			3.4 (1.2)				
	Primary	87		9 (3.5)					1.1 (0.9)						68			3.9 (1.4)				
	Secondary	103		10.3 (2.3)					1.2 (0.8)						75			4.1 (1.4)				
	Higher secondary	70		11.5 (1.8)					1.3 (0.9)						55			4.5 (1.1)				
	Bachelor’s degree and above	156		12.2 (1.4)					1.3 (0.8)						116			5 (0.9)				
**Occupation^a^**								<.001						.38								<.001
	Currently unemployed	202		10.3 (2.9)					1.2 (0.8)						115			3.8 (1.4)				
	Service holder	134		12 (1.6)					1.3 (0.8)						110			5 (0.7)				
	Farmer	28		7.6 (3.9)					1 (0.9)						24			4 (1.5)				
	Businessperson	57		10.6 (2.4)					1.4 (0.8)						55			4.6 (1.2)				
	Day laborer	52		9.4 (2.9)					1.2 (0.9)						39			3.7 (1.4)				
	Other	19		10.7 (3.5)					1 (0.9)						16			4.3 (1.2)				
**Religion^b^**								.08						<.001								.19
	Muslim	454		10.5 (2.9)					1.2 (0.8)						332			4.4 (1.3)				
	Other	38		11.3 (2.3)					1.7 (0.6)						27			3.6 (1.2)				
**Current** **residence^b^**								<.001						0.28								<.001
	Urban	188		11.7 (1.9)					1.3 (0.8)						131			4.8 (0.8)				
	Rural	304		9.8 (3.1)					1.2 (0.8)						228			4 (1.4)				
**Marital** **status^b^**								<.001						.02								.03
	In a marital relationship	331		10.1 (2.9)					1.2 (0.8)						250			4.2 (1.4)				
	Not in a marital relationship	161		11.5 (2.5)					1.4 (0.8)						109			4.6 (1.1)				
**Family** **size^a^**								.48						.57								.25
	1-3 members	109		10.8 (2.8)					1.2 (0.8)						83			4.4 (1.3)				
	3-6 members	316		10.6 (2.9)					1.3 (0.8)						233			4.3 (1.3)				
	≥7 members	67		10.2 (2.9)					1.3 (0.8)						43			4 (1.5)				
**Earning person in** **household^a^**								.30						.24								. 48
	No earning person	6		12.2 (1.5)					1 (0.9)						3			4.7 (0.6)				
	1 earning person	253		10.5 (2.8)					1.2 (0.8)						179			4.3 (1.3)				
	≥2 earning persons	233		10.6 (3)					1.3 (0.8)						177			4.3 (1.3)				
**Monthly income^a^ (BDT^c^)**								<.001						.13								<.001
	No income	202		10.3 (2.9)					1.3 (0.8)						116			3.9 (1.3)				
	≤ 10000	111		9.2 (3.3)					1 (0.9)						89			4 (1.4)				
	10001-20000	80		11.2 (2.1)					1.4 (0.8)						70			4.8 (1.1)				
	20001-30000	39		12.2 (1.9)					1.3 (0.8)						34			4.6 (1.1)				
	30001-40000	33		12 (1.5)					1.2 (0.9)						25			5 (0.8)				
	> 40000	27		12.5 (2)					1.4 (0.7)						25			4.6 (1.1)				
**Availability of water at** **home^b^**								.11						.02								.09
	Yes	449		10.6 (2.9)					1.3 (0.8)						332			4.3 (1.3)				
	No	43		10.4 (2.1)					1 (0.9)						27			4.9 (1)				
**Division of residence^a^**								<.001						<.001								<.001
	Barishal	30		10.4 (3)					1.2 (0.8)						19			4.4 (1.5)				
	Chattogram	87		11.7 (1.7)					1 (0.9)						62			5 (1.1)				
	Dhaka	120		10.8 (2.2)					1.3 (0.7)						85			4.2 (1.2)				
	Khulna	59		11.8 (1.3)					1.8 (0.5)						53			4.8 (1)				
	Mymensingh	38		11 (2.6)					1.6 (0.8)						29			4.6 (1.7)				
	Rajshahi	69		7.7 (3.6)					0.8 (0.8)						68			3.6 (1.2)				
	Rangpur	59		9.4 (3.6)					1.3 (0.8)						26			3.8 (1.4)				
	Sylhet	30		12.3 (1.6)					1.5 (0.9)						17			3.9 (1.3)				

^a^Kruskal-Wallis test.

^b^Wilcoxon rank sum test.

^c^BDT: Bangladeshi Taka. 1 BDT=US $.012.

### Correlations Between Knowledge, Attitude, and Practice Scores

Table 4 illustrates the Spearman rank correlation coefficients and the statistically significant levels of the coefficients. The test showed positive and statistically strong correlations of knowledge with attitudes, knowledge with practices, and attitudes with practices (*P*<.001).

**Table 4:** Correlations between knowledge, attitude, and practice scores.

Correlation	Spearman rank correlation coefficient	*P* value
Knowledge-Attitude	0.21	<.001
Knowledge-Practice	0.45	<.001
Attitude-Practice	0.27	<.001

### Factors Associated With Knowledge

Model 1 in Table 5 demonstrates the factors associated with knowledge about COVID-19. The unadjusted model showed that several factors, such as age group, sex, education, occupation, current residence, administrative division, marital status, and income level were significantly associated with knowledge on COVID-19; the adjusted model, after removing the confounding effects of the independent variables, showed that education, marital status, family size, monthly income, and administrative divisions were significantly associated with knowledge about COVID-19. The higher age groups (ie, 46-55 years: odds ratio [OR] 0.34, 95% CI 0.17-0.7; 56-65 years: OR 0.42, 95% CI 0.2-0.87; ≥66 years: OR 0.18, 95% CI 0.07-0.46) were more likely to have poor knowledge compared to the reference age group of 25 years or below. Female respondents (OR 0.64; 95% CI 0.44-0.94) were more likely to have poor knowledge compared to their male counterparts. Among the different occupation groups, farmers (OR 0.42, 95% CI 0.16-1.09) and day laborers (OR 0.42, 95% CI 0.2-0.86) were significantly more likely to have lower knowledge compared to those who were not employed. In terms of residence, those living in rural areas had a significantly lower level of knowledge toward COVID-19 (OR 0.44, 95% CI 0.3-0.64) compared to their urban counterparts. According to administrative divisions, the Rajshahi division respondents (OR 0.25, 95% CI 0.09-0.65, and adjusted OR 0.22, 95% CI 0.06-0.73) had a lower level of knowledge compared to the respondents from the reference division, Barishal.

**Table 5:** Association of characteristics with knowledge and attitudes toward COVID-19.

Characteristic			Model 1: Knowledge				Model 2: Attitudes		
			OR^a^ (95% CI)		Adjusted OR (95% CI)		OR (95% CI)		Adjusted OR (95% CI)
**Age group (years)**									
	≤25	Ref^b^		Ref		Ref		Ref	
	26-35	1.77* (1.05-3.01)		1.93 (0.92-4.06)		0.98 (0.58-1.64)		0.95 (0.48-1.89)	
	36-45	0.57 (0.3-1.05)		0.79 (0.31-1.99)		0.98 (0.53-1.79)		1.15 (0.5-2.61)	
	46-55	0.34* (0.17-0.7)		0.87 (0.31-2.39)		0.75 (0.38-1.47)		0.74 (0.3-1.8)	
	56-65	0.42* (0.2-0.87)		1.33 (0.45-3.9)		0.66 (0.33-1.31)		0.66 (0.25-1.72)	
	≥66	0.18* (0.07-0.46)		0.38 (0.1-1.41)		1.05 (0.5-2.24)		0.79 (0.28-2.26)	
**Sex**									
	Male	Ref		Ref		Ref		Ref	
	Female	0.64* (0.44-0.94)		0.67 (0.38-1.2)		0.87 (0.6-1.27)		0.9 (0.54-1.5)	
**Education level**									
	No education	Ref		Ref		Ref		Ref	
	Primary	2.38* (1.08-5.25)		1.88 (0.75-4.69)		0.77 (0.42-1.43)		0.74 (0.35-1.56)	
	Secondary	2.43* (1.13-5.23)		1.59 (0.62-4.06)		0.8 (0.44-1.44)		0.56 (0.26-1.2)	
	Higher secondary	7.88* (3.56-17.45)		4.58* (1.53-13.72)		1.19 (0.62-2.29)		0.57 (0.22-1.5)	
	Bachelor’s degree and above	15.53* (7.49-32.2)		6.07* (2.14-17.23)		0.92 (0.53-1.6)		0.3* (0.12-0.77)	
**Occupation**									
	Currently unemployed	Ref		Ref		Ref		Ref	
	Service holder	2.98* (1.89-4.69)		0.77 (0.27-2.2)		1.16 (0.75-1.8)		2.2 (0.83-5.81)	
	Farmer	0.42 (0.16-1.09)		0.62 (0.16-2.45)		0.73 (0.33-1.63)		1.58 (0.46-5.42)	
	Businessperson	1.22 (0.67-2.2)		0.43 (0.13-1.39)		1.66 (0.92-3.02)		3.54* (1.2-10.47)	
	Day laborer	0.42* (0.2-0.86)		0.35 (0.11-1.14)		1.04 (0.57-1.92)		2.37 (0.85-6.61)	
	Other	1.73 (0.67-4.45)		1.15 (0.29-4.6)		0.82 (0.32-2.12)		1.85 (0.48-7.19)	
**Religion**									
	Muslim	Ref		Ref		Ref		Ref	
	Other	1.6 (0.82-3.11)		0.8 (0.29-2.2)		5.15* (2.22-11.93)		3.56* (1.31-9.64)	
**Current residence**									
	Urban	Ref.		Ref.		Ref.		Ref.	
	Rural	0.44* (0.3-0.64)		0.67 (0.39-1.14)		0.87 (0.61-1.26)		0.77 (0.47-1.27)	
**Marital status**									
	In a marital relationship	Ref		Ref		Ref		Ref	
	Not in a marital relationship	2.6* (1.76-3.82)		1.98* (1.14-3.43)		1.4 (0.96-2.05)		1.56 (0.95-2.57)	
**Family size**									
	1-3 members	Ref		Ref		Ref		Ref	
	3-6 members	0.98 (0.63-1.51)		2.13* (1.14-3.96)		1.32 (0.85-2.05)		1.49 (0.86-2.61)	
	>7 members	0.56 (0.3-1.05)		0.98 (0.39-2.47)		1.16 (0.63-2.14)		1.27 (0.57-2.83)	
**Earning person in household**									
	None	Ref		Ref		Ref		Ref	
	1	0.38 (0.07-2.1)		0.23 (0.03-1.81)		1.72 (0.31-9.56)		2.95 (0.46-18.95)	
	≥2	0.42 (0.07-2.32)		0.23 (0.03-1.82)		2.2 (0.39-12.24)		4.03 (0.61-26.56)	
**Monthly income (BDT^c^)**									
	No income	Ref		Ref		Ref		Ref	
	≤ 10000	0.81 (0.5-1.33)		2.2 (0.82-5.92)		0.74 (0.46-1.18)		0.42 (0.17-1.03)	
	10001-20000	1.6 (0.95-2.7)		1.56 (0.54-4.5)		1.34 (0.79-2.25)		0.37 (0.14-1.01)	
	20001-30000	4.5* (2.12-9.56)		2.51 (0.74-8.54)		1.1 (0.55-2.17)		0.29* (0.09-0.91)	
	30001-40000	3.53* (1.62-7.7)		2.73 (0.78-9.58)		1.11 (0.53-2.31)		0.32 (0.1-1.06)	
	>40000	10.16* (3.38-30.52)		14.28* (3.08-66.16)		1.12 (0.5-2.5)		0.25* (0.07-0.9)	
**Availability of water at home**									
	Yes	Ref		Ref		Ref		Ref	
	No	0.51 (0.26-1.01)		0.54 (0.24-1.24)		0.53 (0.27-1.02)		0.54 (0.25-1.15)	
**Knowledge about COVID-19**									
	Poor	N/A^d^		N/A		Ref		Ref	
	Good	N/A		N/A		1.93* (1.34-2.76)		1.76* (1.07-2.89)	
**Division of residence**									
	Barishal	Ref		Ref		Ref		Ref	
	Chattogram	1.4 (0.61-3.23)		1.38 (0.48-3.94)		1.06 (0.45-2.47)		1.14 (0.46-2.84)	
	Dhaka	0.7 (0.31-1.59)		1.02 (0.36-2.91)		1.11 (0.49-2.51)		0.86 (0.35-2.12)	
	Khulna	2.37 (0.96-5.81)		1.58 (0.51-4.89)		7.35* (2.71-19.94)		8.96* (2.98-26.98)	
	Mymensingh	2.01 (0.76-5.3)		2.87 (0.83-9.94)		4.2* (1.5-11.73)		4.29* (1.39-13.27)	
	Rajshahi	0.25* (0.09-0.65)		0.22* (0.06-0.73)		0.45 (0.18-1.14)		0.42 (0.15-1.18)	
	Rangpur	0.84 (0.34-2.04)		3.41 (0.98-11.83)		1.35 (0.56-3.3)		1.54 (0.53-4.49)	
	Sylhet	5.23* (1.66-16.51)		5.37* (1.24-23.22)		3.5* (1.2-10.2)		1.63 (0.49-5.42)	

^a^OR: odds ratio.

^b^Ref: reference.

^c^BDT: Bangladeshi Taka. 1 BDT=US $.012.

^d^N/A: not applicable.

*P<.05.

**Figure 2:**
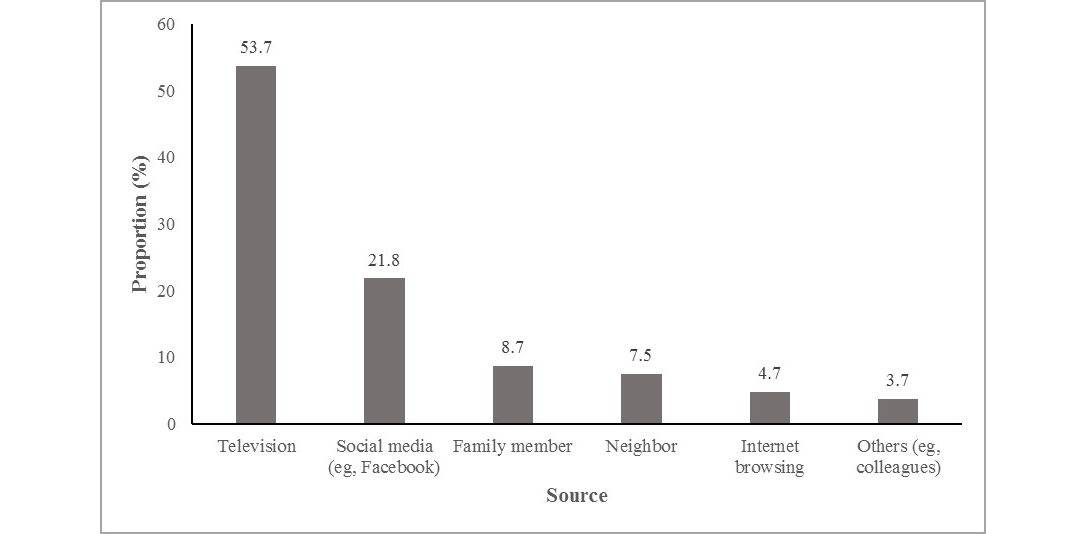
Source of knowledge about COVID-19 among the respondents.

Figure 2 shows the sources of knowledge and indicates that television (264/492, 53.7%), followed by social media (107/492; 21.8%), is the major source of knowledge on COVID-19 for the study population. Other important sources included family members (43/492, 8.7%), neighbors (37/492, 7.5%), and the internet (23/492, 4.7%).

### Factors Associated With Attitudes

Model 2 of Table 5 demonstrates the factors associated with attitudes toward COVID-19. In the unadjusted model, religion, knowledge about COVID-19, and administrative division showed a significant association with a positive attitude toward COVID-19. Controlling for the confounding effects of the independent variables confirmed the significant influence of education, occupation, religion, monthly income, knowledge about COVID-19, and administrative division on positive attitudes toward COVID-19. According to education level, people with higher education levels (adjusted OR 0.3, 95% CI 0.12-0.77) were more likely to have a negative attitude toward control of COVID-19 than those with no education. People with an income level between BDT 20,001 and 30,000 (US $198 and $297, adjusted OR 0.29, 95% CI 0.09-0.91) and more than BDT 40,000 (US $396, adjusted OR 0.25, 95% CI 0.07-0.9) had a higher likelihood of a negative attitudes toward controlling COVID-19 compared to those with no income.

**Figure 3:**
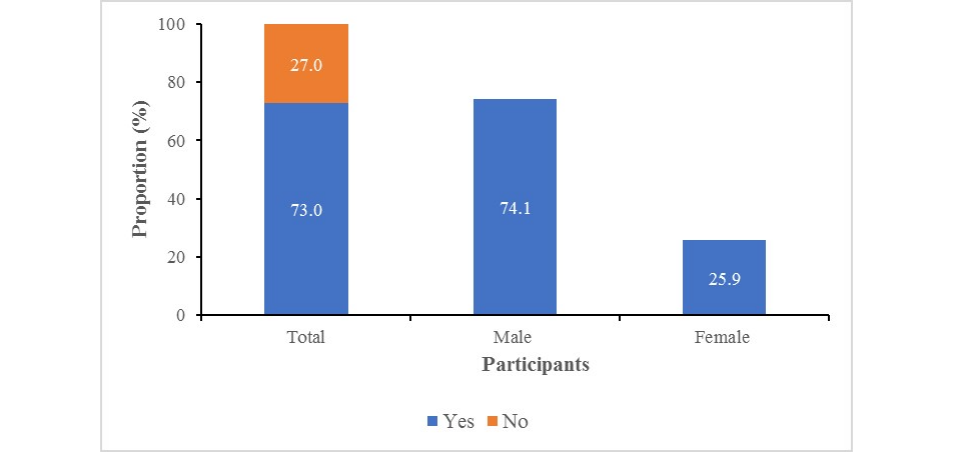
Distribution of respondents according to their mobility during the lockdown period.

Figure 3 shows that 359 of the 492 study participants (73%) went outside the home in the week preceding the survey during the lockdown period. As mentioned earlier in the *Methods* section, information on practices toward COVID-19 was collected only for those respondents who left their homes during the lockdown period. Among this group, 266 of 359 (74.1%) were male.

**Figure 4:**
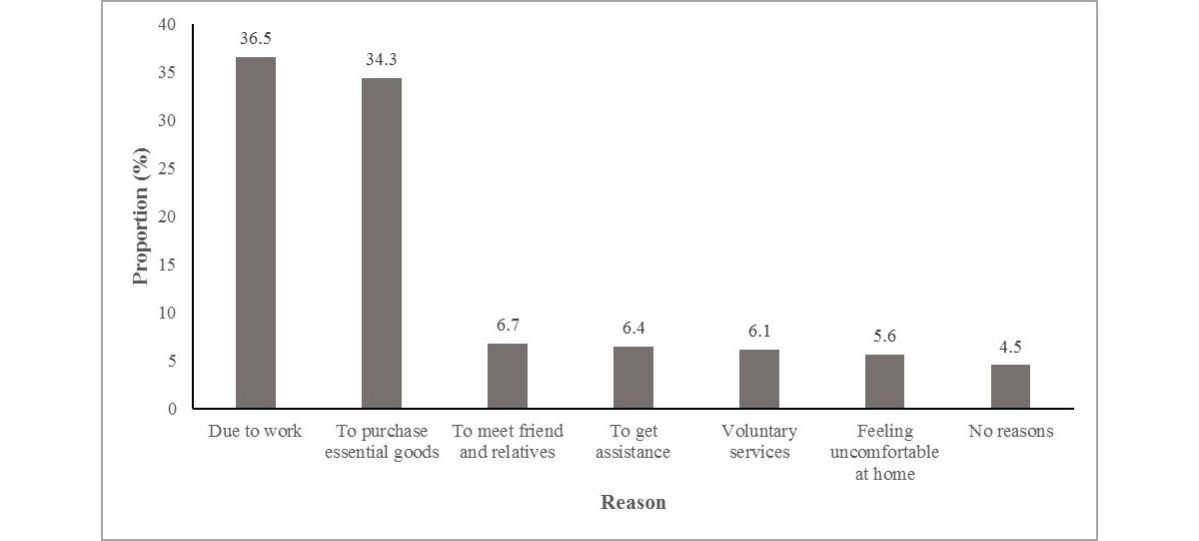
Reason for going outside of the home among respondents during the lockdown period.

Figure 4 shows that among the 359 respondents who left their homes during the lockdown, 131 (37.5%) went out due to work, followed by 123 (34.3%) to purchase essential goods such as food and medicine. However, 16 of the 359 respondents (4.5%) went out for no particular reason.

### Factors Associated With Practices

Table 6 demonstrates the factors associated with self-reported practices regarding COVID-19. The unadjusted model showed that several sociodemographic factors, such as age group, education, occupation, monthly income, water availability at home, knowledge, attitude, and administrative division were significantly associated with good practices toward COVID-19. The adjusted model, after removing the other variables' confounding effects, showed that education level, occupation, religion, monthly income, knowledge, attitude, and administrative divisions were significantly associated with good practices toward COVID-19. The results showed that compared to Muslims, people of other religions (adjusted OR 0.14; 95% CI 0.04-0.51) had a higher likelihood of poor practices. According to income level, respondents with a monthly income of more than BDT 40,000 (US $396; adjusted OR 0.1; 95% CI 0.02-0.6) had more likelihood of poor practices than those in the no income reference category. Respondents who were from the Rajshahi division (OR 0.18; 95% CI 0.06-0.59) were more likely to have poor practices compared to those from the reference Barisal division.

**Table 6:** Association of characteristics with self-reported practices toward COVID-19.

Characteristic			n		Unadjusted OR^a^ (95% CI)		Adjusted OR (95% CI)
**Age group (years)**							
	≤25	58		Ref^b^		Ref	
	26-35	134		2.06* (1.1-3.85)		1.52 (0.56-4.12)	
	36-45	69		1.57 (0.77-3.18)		1.21 (0.36-4)	
	46-55	46		0.98 (0.44-2.16)		1.93 (0.5-7.41)	
	56-65	34		0.73 (0.3-1.77)		2.27 (0.55-9.4)	
	≥66	18		0.3 (0.08-1.17)		0.52 (0.08-3.19)	
**Sex**							
	Male	266		Ref		Ref	
	Female	93		0.69 (0.43-1.12)		0.92 (0.39-2.18)	
**Education level**							
	No education	45		Ref		Ref	
	Primary	68		1.46 (0.61-3.5)		1.03 (0.33-3.22)	
	Secondary	75		2.9* (1.26-6.7)		2.75 (0.86-8.82)	
	Higher secondary	55		2.92* (1.21-7.04)		1.89 (0.49-7.22)	
	Bachelor’s degree and above	116		7.18* (3.22-16.03)		7.43* (1.95-28.33)	
**Occupation**							
	Currently unemployed	115		Ref		Ref	
	Service holder	110		2.51* (1.47-4.3)		1.99 (0.42-9.47)	
	Farmer	24		0.97 (0.39-2.4)		7.34* (1.09-49.33)	
	Businessperson	55		1.67 (0.87-3.2)		2.39 (0.45-12.75)	
	Day laborer	39		0.72 (0.33-1.56)		3.47 (0.67-18.09)	
	Other	16		1.26 (0.44-3.61)		1.3 (0.22-7.86)	
**Religion**							
	Muslim	332		Ref		Ref	
	Other	27		0.46 (0.2-1.08)		0.14* (0.04-0.51)	
**Current residence**							
	Urban	131		Ref		Ref	
	Rural	228		0.71 (0.46-1.09)		0.94 (0.46-1.93)	
**Marital status**							
	In a marital relationship	250		Ref		Ref	
	Not in a marital relationship	109		1.07 (0.68-1.68)		0.89 (0.43-1.85)	
**Family size**							
	1-3 members	83		Ref		Ref	
	3-6 members	233		1.04 (0.63-1.72)		0.82 (0.38-1.8)	
	≥7 members	43		0.67 (0.31-1.42)		0.68 (0.21-2.25)	
**Earning person in household**							
	None	3		Ref.		Ref.	
	1 person	179		0.51 (0.05-5.68)		0.21 (0.01-3.6)	
	≥2 persons	177		0.37 (0.03-4.13)		0.19 (0.01-3.15)	
**Monthly income (BDT^c^)**							
	No income	116		Ref		Ref	
	≤10000	89		0.65 (0.36-1.16)		0.32 (0.08-1.33)	
	10001-20000	70		2.4* (1.3-4.41)		0.9 (0.21-3.8)	
	20001-30000	34		1.79 (0.83-3.88)		0.32 (0.06-1.74)	
	30001-40000	25		3.0*1 (1.2-7.54)		0.85 (0.13-5.77)	
	>40000	25		1.11 (0.47-2.66)		0.1* (0.02-0.6)	
**Availability of water at home**							
	Yes	332		Ref		Ref	
	No	27		2.46* (1.07-5.63)		2.27 (0.68-7.6)	
**Knowledge about COVID-19**							
	Poor	195		Ref		Ref	
	Good	164		3.13* (2.03-4.83)		2.33* (1.16-4.68)	
**Attitude toward COVID-19**							
	Negative	96		Ref		Ref	
	Positive	263		2.43* (1.59-3.72)		3.87* (1.95-7.68)	
**Division of residence**							
	Barishal	19		Ref		Ref	
	Chattogram	62		6.38* (2.08-19.53)		11.85* (2.72-51.57)	
	Dhaka	85		0.87 (0.32-2.4)		1.5 (0.4-5.63)	
	Khulna	53		6.72* (2.11-21.42)		10.09* (2.15-47.41)	
	Mymensingh	29		1.47 (0.46-4.73)		0.89 (0.2-4.03)	
	Rajshahi	68		0.18* (0.06-0.59)		0.32 (0.07-1.42)	
	Rangpur	26		0.33 (0.09-1.24)		0.48 (0.09-2.74)	
	Sylhet	17		0.29 (0.06-1.38)		0.24 (0.04-1.51)	

^a^OR: odds ratio.

^b^Ref: reference.

^c^BDT: Bangladeshi Taka. 1 BDT=US $.012.

*P<.05.

## Discussion

### Principal Results

Appropriate participation is essential for effective control and prevention of an infectious disease such as COVID-19. Nevertheless, this participation relies fundamentally on adequate knowledge and improved practices against the infection. In our study, we applied a survey through one-to-one audio communication instead of web-based surveys to evaluate gaps and needs in the fight against COVID-19 during the period of its rapid rise in Bangladesh. We found that 55.5% of respondents had poor knowledge, 49% expressed a negative attitude about controlling this disease nationally and globally, and 53.5% had unfavorable practices toward COVID-19. Our analysis has shown that the knowledge related to COVID-19 of certain demographic and socioeconomic groups (eg, those who were aged ≥46 years; were female; had no education; were farmers, day laborers, or rural residents; were in a marital relationship; had a larger family; earned less than BDT 20,001 [US $236]; and were residents of Rajshahi Division) is significantly lower than that of respondents in the corresponding reference categories. Most people rely on television, followed by social media, as a source of knowledge. Almost three-quarters of the respondents left home during the lockdown period, the majority of whom were male (74%). Those with no education lagged behind those with secondary or higher education. Finally, we found that better practice of COVID-19 health measures was associated with good knowledge and a positive attitude toward COVID-19.

We also found poor knowledge to be significantly associated with several demographic and socioeconomic factors of individuals. Better socioeconomic status contributed to a higher level of COVID-19–related knowledge among people in Bangladesh. Although the study was conducted some months after the onset of the pandemic, the influence of socioeconomic status remained, indicating unequal penetration of information on COVID-19 management throughout society. The study findings indicate that knowledge about COVID-19 increased as age decreased; older people had lower levels of knowledge. This finding is supported by several other international studies from developing and developed countries that report older respondents having poor knowledge of COVID-19 compared to younger populations [10,21,25]. The physical condition, cognitive status, and limited access to information of the older population may influence their ability to understand information on COVID-19 and may contribute to the poorer knowledge level among this population [26]. Lack of familiarity and limited use of modern technology may be other reasons for poor knowledge among older adults [27,28]. The study found that farmers and daily laborers were more likely to have poor knowledge about COVID-19. This finding is similar to that in a study conducted in China [21], which found that laborers had poorer knowledge of this topic.

From the beginning of the pandemic, there has never been an effective lockdown in place in Bangladesh. Primarily, this may have been the result of the government’s policy to declare this crucial time a general holiday rather than a lockdown [29,30]. The notion of a holiday did not help people to recognize the importance of staying at home as much as possible. People were under the impression that they were free to roam around. As a result, many people willingly ignored the stay-at-home or social distancing guidelines. They took the opportunity to move to different cities across the country, which we anticipate might have contributed to the rapid spread of infection at the community level throughout the country [31]. Subsequently, the government extended the general holiday without ensuring adequate subsistence support for low-income residents before imposing a lockdown, which may also have compelled people to leave their homes [32]. Further, the rapid change in lockdown timing every week might have precluded people from making adequate preparation. Moreover, the government’s inability to provide information on how people in lockdown situations could access essential materials for their everyday life and engage community groups for meeting essential needs may be one reason for the poor practice of safety measures among the population [33]. This result reinforces the conclusions of previous studies identifying strict prevention practices and mobilizing community volunteers to support people under lockdown as the primary solutions for reducing the spread of COVID-19 in China and Vietnam [21,34,35].

Bangladesh is still a predominantly rural country, with only 37% of its population living in urban areas [36]. However, rural areas lag behind urban areas in most socioeconomic and health indicators. For example, only 76% of rural areas are served by the national electricity grid (compared to 92% of urban areas), and only 38% of rural households own a television (compared to 70% in urban areas). In terms of demographic and health indicators, we find that 61% of rural women marry before the age of 18 years (compared to 55% of urban women), 79% of rural women receive antenatal care from a skilled provider (compared to 90% of urban women), and 45% give birth in a health facility (compared to 63% of urban women); moreover, 33% of rural children under the age of 5 years are stunted (compared to 25% of urban children) [37]. Consistent with this reduced health-seeking behavior, the current study found that rural residents were less likely to adhere to COVID-19–related hygiene practices. Adding to this poor practice was the risk of the rapid spread of infection caused by migrants returning to the rural community from different countries around the world during the pandemic period [38,39]. Lower awareness in rural communities about health policy and programs has been observed in several other countries, and this phenomenon may pose a higher degree of threat in the case of communicable diseases such as COVID-19 [10,40,41].

Good knowledge and a positive attitude toward controlling COVID-19 were found to be associated with good practice of safety measures. This finding is well recognized in several global studies, in which it was found that good knowledge and a positive attitude toward COVID-19 lead to improved practice of safety measures [10,22,42,43]. It is worth mentioning that the consistency of theory-based approaches demonstrates an association among knowledge, belief, and change in human behavior [44]. Thus, it has been recognized that adequate and proper knowledge of a specific health emergency is a key modifier of personal belief that leads to changing human behavior [45]. When combined with good knowledge and positive attitudes, a higher level of appropriate practice was identified among respondents. This may result from a higher level of accurate knowledge about common symptoms, mode of spread, and knowledge about preventive practices (eg, avoiding crowded places, maintaining recommended social distance, and frequent hand washing).

Because KAP levels vary across different socioeconomic groups, we recommend that customized information on COVID-19 be developed targeting different groups in Bangladesh, such as villagers, slum-dwellers, township residents, and urban middle-class citizens. Special emphasis should be given to the groups with lower KAP scores, such as older persons, females, less educated people, farmers, day laborers, rural residents, those in a marital relationship, those with a larger family, those with low income, and residents of the Rajshahi division. The information should be clearly and widely circulated through contextually appropriate channels at a massive level, emphasizing television and social media, as these were found to be the major sources of information. Additionally, because many people did not comply with the lockdown directives, the implementation of a lockdown should encompass a well-planned support system that includes subsistence support for low-income individuals, arranging emergency requirements for the locked-down community, communicating clearly what to do and what not to do during the lockdown, and clarifying whom to consult in the case of any unforeseen situation. A voluntary community support group might be engaged to support people’s needs. Instead of arbitrarily increasing the duration of lockdown on a week-by-week basis, a predetermined guide period should be established, in consultation with epidemiologists, and clearly communicated to the public so that they may make adequate preparation to stay at home for the entire period. The term “national holiday” may not convey the right message to the public; therefore, “lockdown” or any contextually appropriate synonym, in consultation with communication experts or social scientists, should be used instead. Special attention should be directed toward rural communities, where engagement in COVID-19 health practices is lower. Finally, because practices are associated with knowledge and attitude, we recommend that a scientifically oriented social and behavioral change communication strategy be developed in consultation with the relevant experts. Religious, cultural, political, and other community-based agents can be consulted and actively engaged in turning these strategies into actions or practices.

### Limitations

This study is not free from limitations. First, the study is limited by the sampling method. Thus, selection bias may exist. Another limitation is the study population. This study only considered adults who use mobile phones. Moreover, data were collected over mobile phone calls, which is not a familiar process in Bangladesh. Thus, response bias may exist, and only those concerned more about the COVID-19 emergency may have participated in the study. Therefore, the results may not be generalizable to other people who are not mobile phone users. The number of questions under the Attitudes section, where only 2 questions were considered in the KAP questionnaire to measure the attitude level, limited the depth of this domain. The major limitation can be considered with regard to the study design. As this is a cross-sectional study, causal inferences cannot be drawn between the significant factors and the KAP levels.

### Strengths

Despite the above limitations, the study’s strength lies in the fact that it includes data from 8 administrative divisions throughout the country and that participants from both rural and urban areas were surveyed by telephone. The findings of the study, to our understanding, will motivate and alert policy makers and program implementers who are working on appropriate health education, risk communication, community engagement, and social and behavioral change communication strategies based on levels of KAP toward COVID-19.

Further research is needed to understand the KAP of service providers in COVID-19 pandemic response. Qualitative formative research will aid the design of effective communication strategies to address the pandemic, and subsequent implementation and evaluation research can generate useful knowledge about the implementation and scaleup of such strategies in different parts of Bangladesh and other countries facing similar KAP-related challenges.

### Conclusions

Risk communication and community engagement are an integral part of pandemic management [46]. For countries such as Bangladesh, where most of the community lacks proper health practices and where timely information penetration is unequal, KAP studies such as this one can support public health decision-makers in designing evidence-informed and context-specific risk communication and behavioral change communication strategies. These evidence-informed strategies may help in managing health emergencies such as the COVID-19 pandemic for people from all socioeconomic groups.

## Appendix

Multimedia Appendix 1 Study questionnaire.

Multimedia Appendix 2 Study data set.

Multimedia Appendix 3 Cross-tabulation of demographic and socioeconomic characteristics of respondents by administrative division.

Multimedia Appendix 4 Response of questions regarding knowledge, attitudes, and practices toward COVID-19.

## Abbreviations

KAP: knowledge, attitudes, and practices
OR: odds ratio
WHO: World Health Organization
