# Supplementary material for: COVID-19 Knowledge, Attitudes, and Practices Among People in Bangladesh: Telephone-Based Cross-sectional Survey
Source: JMIR Form Res. 2021 Nov 5;5(11):e28344. doi: 10.2196/28344 (PMC8575001; doi:10.2196/28344)
Supplement: Multimedia Appendix 1 [file formative_v5i11e28344_app1.docx]

# Questionnaire

**Study Title:** Knowledge, Attitude and Practice Towards COVID-19 Among People in Bangladesh: A Telephonic Cross-sectional Survey.

**Date:** ___/___/2020 **Questionnaire ID:** |___|___|___|___|

**Section A: Demographic and Socioeconomic Information.**

1. **Name of respondents:** ____________________________________________________________
2. **Contact information:** ____________________________________________________________
3. **Age: __________________ years**
4. **Gender:**

1. Male

2. Female

1. **Education:**
2. No education
3. Primary (1-5)
4. Secondary (6-10)
5. HSC/ equivalent (11-12)
6. Higher (above 12)
7. **Occupation**
8. Farmar
9. Business/ informal worker
10. Service holder – government job
11. Service holder - private job
12. Housewife
13. Day labor
14. Driver (Rickshaw/ autorickshaw/ CNG/ Bus)
15. Student
16. Retired/ aged people
17. Unemployed
18. Others _______________________
19. **Religion**
20. Islam
21. Hinduism
22. Buddhism
23. Christian
24. Others
25. **Marital status**
26. Married
27. Unmarried
28. Widow
29. Separate/ Divorce
30. **Number of family member/ Family size: _____________________________.**
31. **Current residence**
32. Urban
33. Rural
34. **Number of rooms in current residence/ home: ________________________.**
35. **Number of persons in current residence/ home: _______________________.**
36. **Running water supply is available currently**
37. Yes
38. No
39. **Number of toilets at home: __________________.**
40. **Number of earning person in the family: _______.**
41. **Monthly income of respondent: ______________Bangladeshi TK (currency).**
42. **Division**
43. Barishal
44. Chattogram
45. Dhaka
46. Khulna
47. Mymensingh
48. Rajshahi
49. Rangpur
50. Sylhet

**Section B: Knowledge, Attitude, and Practice against Coronavirus Disease 2019 (COVID-19).**

**Knowledge (* indicates the correct response):**

1. The main clinical symptoms of COVID-19 are fever, dry cough, and shortness of breath.

| 1. Yes* | 1. No | 1. Don’t know |
| --- | --- | --- |

1. Neck pain, sore throat, tiredness, runny nose, sneezing, and diarrhea are less common in persons infected with the COVID-19.

| 1. Yes* | 1. No | 1. Don’t know |
| --- | --- | --- |

1. Currently, there is no effective treatment for COVID-2019 disease, but early symptomatic and supportive treatment can help most patients recover from the infection.

| 1. Yes* | 1. No | 1. Don’t know |
| --- | --- | --- |

1. Not all persons with COVID-2019 will develop severe cases. Only those who are elderly, have chronic illnesses such as diabetes, high BP, heart disease, etc. are more likely to be in severe cases.

| 1. Yes* | 1. No | 1. Don’t know |
| --- | --- | --- |

1. Eating or contacting infected wild animals would result in the infection by the virus.

| 1. Yes* | 1. No | 1. Don’t know |
| --- | --- | --- |

1. Persons with COVID-2019 can infect the virus to others when a fever is not present.

| 1. Yes* | 1. No | 1. Don’t know |
| --- | --- | --- |

1. The COVID-19 virus spreads via respiratory droplets of infected individuals.

| 1. Yes* | 2. No | 1. Don’t know |
| --- | --- | --- |

1. It is necessary for all to take measures to prevent the infection by the COVID-19 virus.

| 1. Yes* | 1. No | 1. Don’t know |
| --- | --- | --- |

1. To prevent the infection by COVID-19, individuals should avoid going to crowded places such as market, and avoid taking public transportations.

| 1. Yes* | 1. No | 1. Don’t know |
| --- | --- | --- |

1. At least 1 meter/ 3 feet is the recommended social distance or physical distance for COVID-19 if go outside of the home.

| 1. Yes* | 1. No | 1. Don’t know |
| --- | --- | --- |

1. To prevent the infection by COVID-19, an individual should wash hands frequently after coming from outside, before eating or touching the mouth, nose, or eyes.

| 1. Yes* | 1. No | 1. Don’t know |
| --- | --- | --- |

1. To prevent the spread of COVID-19, the recommended time for washing hands with soap/ alcohol is a minimum of 20-30 seconds.

| 1. Yes* | 1. No | 1. Don’t know |
| --- | --- | --- |

1. Isolation and treatment of people who are infected with the COVID-19 virus are effective ways to reduce the spread of the virus.

| 1. Yes* | 1. No | 1. Don’t know |
| --- | --- | --- |

1. People who have contact with someone infected with the COVID-19 virus should be immediately isolated in a proper place. In general, the observation period is 14 days.

| 1. Yes* | 1. No | 1. Don’t know |
| --- | --- | --- |

1. What is the main source of your knowledge about Coronavirus?
2. Television
3. Mobile (SMS/ voice call)
4. Internet browsing
5. Social media i.e., Facebook, YouTube, etc.
6. Local micking
7. Newspaper
8. Family member
9. Neighbor
10. Others: ____________________________.

**Attitudes:**

1. I agree that COVID-19 will finally be successfully controlled across the world.

| 1. Yes | 1. No | 1. Don’t know |
| --- | --- | --- |

1. I have confidence that Bangladesh will win the battle against the COVID-19.

| 1. Yes | 1. No | 1. Don’t know |
| --- | --- | --- |

**Practices:**

1. I have gone outside of the home during the lockdown period?

| 1. Yes | 1. No |
| --- | --- |

1. I have gone out of home _______ times.
2. I have gone out of home during the COVID-19 outbreaks due to ____________________________
3. Official work
4. Purchase food/medicine
5. To get relief/assistance
6. To earn money to survive
7. Feeling uncomfortable at home
8. No enough space at home
9. To meet friend and relatives
10. Voluntary services
11. No reasons
12. Others, please write: ____________________________________________________
13. When I went out, I have avoided the crowded place.

| 1. Yes | 1. No | 1. Sometimes |
| --- | --- | --- |

1. When I went out, I have maintained the recommended social distance of 1 meter or 3 feet.

| 1. Yes | 1. No | 1. Sometimes |
| --- | --- | --- |

1. When I went out, I have worn a mask regularly and thoroughly.

| 1. Yes | 1. No | 1. Sometimes |
| --- | --- | --- |

1. If I were to go out, I have washed my hand after coming from outside and before eating or touching mouth, nose, or eyes regularly and thoroughly.

| 1. Yes | 1. No | 1. Sometimes |
| --- | --- | --- |

1. I have maintained the recommended hand washing time of 20-30 seconds regularly and thoroughly.

| 1. Yes | 1. No | 1. Sometimes |
| --- | --- | --- |

Thank you so much for your time!
