# Supplementary material for: COVID-19 Knowledge, Attitudes, and Practices Among People in Bangladesh: Telephone-Based Cross-sectional Survey
Source: JMIR Form Res. 2021 Nov 5;5(11):e28344. doi: 10.2196/28344 (PMC8575001; doi:10.2196/28344)
Supplement: Multimedia Appendix 3 [file formative_v5i11e28344_app3.docx]

**Supplemental Table 1:** Demographic and socioeconomic characteristics of respondents by administrative divisions.

| **Characteristics** | **Administrative Division** | | | | | | | | **Total n (%)**  **(N=492)** |
| --- | --- | --- | --- | --- | --- | --- | --- | --- | --- |
|  | **Barishal** | **Chattogram** | **Dhaka** | **Khulna** | **Mymensingh** | **Rajshahi** | **Rangpur** | **Sylhet** |  |
| **Age groups** |  |  |  |  |  |  |  |  |  |
| ≤ 25 | 7(8.33) | 21(25) | 11(13.1) | 6(7.14) | 5(5.95) | 18(21.43) | 2(2.38) | 14(16.67) | 84(17.07) |
| 26-35 | 10(5.81) | 26(15.12) | 41(23.84) | 32(18.6) | 23(13.37) | 23(13.37) | 6(3.49) | 11(6.4) | 172(34.96) |
| 36-45 | 6(7.23) | 19(22.89) | 27(32.53) | 13(15.66) | 3(3.61) | 8(9.64) | 4(4.82) | 3(3.61) | 83(16.87) |
| 46-55 | 4(6.78) | 13(22.03) | 16(27.12) | 4(6.78) | 2(3.39) | 11(18.64) | 8(13.56) | 1(1.69) | 59(11.99) |
| 56-65 | 2(3.7) | 4(7.41) | 13(24.07) | 3(5.56) | 2(3.7) | 9(16.67) | 21(38.89) | - | 54(10.98) |
| ≥ 66 | 1(2.5) | 4(10) | 12(30) | 1(2.5) | 3(7.5) | - | 18(45) | 1(2.5) | 40(8.13) |
| **Sex** |  |  |  |  |  |  |  |  |  |
| Male | 24(7.48) | 61(19) | 67(20.87) | 38(11.84) | 31(9.66) | 48(14.95) | 31(9.66) | 21(6.54) | 321(65.24) |
| Female | 6(3.51) | 26(15.2) | 53(30.99) | 21(12.28) | 7(4.09) | 21(12.28) | 28(16.37) | 9(5.26) | 171(34.76) |
| **Education Level** |  |  |  |  |  |  |  |  |  |
| No education | 3(3.95) | 2(2.63) | 38(50) | 2(2.63) | 5(6.58) | 3(3.95) | 23(30.26) | - | 76(15.45) |
| Primary | 6(6.9) | 12(13.79) | 21(24.14) | 4(4.6) | 7(8.05) | 21(24.14) | 14(16.09) | 2(2.3) | 87(17.68) |
| Secondary | 7(6.8) | 18(17.48) | 25(24.27) | 9(8.74) | 7(6.8) | 18(17.48) | 14(13.59) | 5(4.85) | 103(20.93) |
| Higher secondary | 2(2.86) | 17(24.29) | 11(15.71) | 11(15.71) | 4(5.71) | 14(20) | 3(4.29) | 8(11.43) | 70(14.23) |
| Bachelor and above | 12(7.69) | 38(24.36) | 25(16.03) | 33(21.15) | 15(9.62) | 13(8.33) | 5(3.21) | 15(9.62) | 156(31.71) |
| **Occupation** |  |  |  |  |  |  |  |  |  |
| Currently not employed | 12(5.94) | 42(20.79) | 47(23.27) | 17(8.42) | 9(4.46) | 31(15.35) | 28(13.86) | 16(7.92) | 202(41.06) |
| Service holder | 8(5.97) | 23(17.16) | 35(26.12) | 29(21.64) | 13(9.7) | 12(8.96) | 7(5.22) | 7(5.22) | 134(27.24) |
| Farmer | 4(14.29) | 6(21.43) | 1(3.57) | 1(3.57) | 3(10.71) | 5(17.86) | 8(28.57) | - | 28(5.69) |
| Businessman | 3(5.26) | 10(17.54) | 11(19.3) | 7(12.28) | 10(17.54) | 8(14.04) | 3(5.26) | 5(8.77) | 57(11.59) |
| Day labor | 2(3.85) | 3(5.77) | 20(38.46) | 1(1.92) | 3(5.77) | 9(17.31) | 13(25) | 1(1.92) | 52(10.57) |
| Others (e.g., beggar, hawker) | 1(5.26) | 3(15.79) | 6(31.58) | 4(21.05) | - | 4(21.05) | 0(0) | 1(5.26) | 19(3.86) |
| **Religion** |  |  |  |  |  |  |  |  |  |
| Muslim | 28(6.17) | 85(18.72) | 114(25.11) | 45(9.91) | 37(8.15) | 69(15.2) | 59(13) | 17(3.74) | 454(92.28) |
| Others | 2(5.26) | 2(5.26) | 6(15.79) | 14(36.84) | 1(2.63) | - | - | 13(34.21) | 38(7.72) |
| **Current residence** |  |  |  |  |  |  |  |  |  |
| Urban | 13(6.91) | 33(17.55) | 74(39.36) | 23(12.23) | 10(5.32) | 14(7.45) | 6(3.19) | 15(7.98) | 188(38.21) |
| Rural | 17(5.59) | 54(17.76) | 46(15.13) | 36(11.84) | 28(9.21) | 55(18.09) | 53(17.43) | 15(4.93) | 304(61.79) |
| **Marital status** |  |  |  |  |  |  |  |  |  |
| In a marital relationship | 24(7.25) | 51(15.41) | 89(26.89) | 43(12.99) | 28(8.46) | 44(13.29) | 41(12.39) | 11(3.32) | 331(67.28) |
| Not in a marital relationship | 6(3.73) | 36(22.36) | 31(19.25) | 16(9.94) | 10(6.21) | 25(15.53) | 18(11.18) | 19(11.8) | 161(32.72) |
| **Family size** |  |  |  |  |  |  |  |  |  |
| 1-3 members | 4(3.67) | 16(14.68) | 31(28.44) | 19(17.43) | 13(11.93) | 13(11.93) | 11(10.09) | 2(1.83) | 109(22.15) |
| 3-6 members | 19(6.01) | 58(18.35) | 75(23.73) | 37(11.71) | 22(6.96) | 48(15.19) | 39(12.34) | 18(5.7) | 316(64.23) |
| 7 & more members | 7(10.45) | 13(19.4) | 14(20.9) | 3(4.48) | 3(4.48) | 8(11.94) | 9(13.43) | 10(14.93) | 67(13.62) |
| **Earning person in family** |  |  |  |  |  |  |  |  |  |
| No earning person | - | 1(16.67) | 1(16.67) | 0(0) | 1(16.67) | - | 2(33.33) | 1(16.67) | 6(1.22) |
| Single earning person | 18(7.11) | 54(21.34) | 60(23.72) | 30(11.86) | 23(9.09) | 26(10.28) | 30(11.86) | 12(4.74) | 253(51.42) |
| Two & more persons | 12(5.15) | 32(13.73) | 59(25.32) | 29(12.45) | 14(6.01) | 43(18.45) | 27(11.59) | 17(7.3) | 233(47.36) |
| **Monthly Income** |  |  |  |  |  |  |  |  |  |
| No income | 13(6.44) | 41(20.3) | 53(26.24) | 13(6.44) | 11(5.45) | 29(14.36) | 26(12.87) | 16(7.92) | 202(41.06) |
| ≤ 10000 | 2(1.8) | 17(15.32) | 19(17.12) | 4(3.6) | 11(9.91) | 30(27.03) | 27(24.32) | 1(0.9) | 111(22.56) |
| 10001-20000 | 5(6.25) | 9(11.25) | 24(30) | 15(18.75) | 14(17.5) | 6(7.5) | 2(2.5) | 5(6.25) | 80(16.26) |
| 20001-30000 | 4(10.26) | 7(17.95) | 8(20.51) | 9(23.08) | 1(2.56) | 2(5.13) | 3(7.69) | 5(12.82) | 39(7.93) |
| 30001-40000 | 3(9.09) | 8(24.24) | 7(21.21) | 10(30.3) | 0(0) | 2(6.06) |  | 3(9.09) | 33(6.71) |
| > 40000 | 3(11.11) | 5(18.52) | 9(33.33) | 8(29.63) | 1(3.7) |  | 1(3.7) | 0(0) | 27(5.49) |
| **Availability of running water at home** |  |  |  |  |  |  |  |  |  |
| Yes | 29(6.46) | 74(16.48) | 112(24.94) | 57(12.69) | 37(8.24) | 67(14.92) | 43(9.58) | 30(6.68) | 449(91.26) |
| No | 1(2.33) | 13(30.23) | 8(18.6) | 2(4.65) | 1(2.33) | 2(4.65) | 16(37.21) | - | 43(8.74) |
