# Supplementary material for: COVID-19 Knowledge, Attitudes, and Practices Among People in Bangladesh: Telephone-Based Cross-sectional Survey
Source: JMIR Form Res. 2021 Nov 5;5(11):e28344. doi: 10.2196/28344 (PMC8575001; doi:10.2196/28344)
Supplement: Multimedia Appendix 4 [file formative_v5i11e28344_app4.docx]

**Supplemental Table 2:** Response of questions regarding knowledge, attitude and practice (KAP) towards COVID-19.

| Knowledge related questions | Yes (Correct), n (%) | No (Incorrect), n (%) | Don't know (Confuse),  n (%) |
| --- | --- | --- | --- |
| 1. Fever, dry cough and shortness of breath are the main clinical symptoms of COVID-19 disease. | 447 (90.85) | 10 (2.03) | 35 (7.11) |
| 2. Neck pain/sore throat, tiredness, runny nose, sneezing and diarrhea are fewer common symptoms of COVID-19 disease. | 272 (55.28) | 132 (26.83) | 88 (17.89) |
| 3. Currently there is no effective treatment except symptomatic and supportive treatment. | 354 (71.95) | 37 (7.52) | 101 (20.53) |
| 4. The elder people with chronic illnesses such as diabetic, high BP, heart disease etc. are more likely to be severe cases. | 358 (72.76) | 48 (9.76) | 86 (17.48) |
| 5. Eating or contacting infected wild animals would result in the infection by the virus. | 296 (60.16) | 68 (13.82) | 128 (26.02) |
| 6. Persons with COVID-2019 without fever can infect others. | 307 (62.4) | 70 (14.23) | 115 (23.37) |
| 7. The COVID-19 virus spreads via respiratory droplets of infected individuals. | 404 (82.11) | 27 (5.49) | 61 (12.4) |
| 8. It is necessary to all to take measures to prevent the infection by the COVID-19 virus. | 276 (56.1) | 163 (33.13) | 53 (10.77) |
| 9. Individuals should avoid going to crowded places such as market, public transportations to prevent the infection. | 409 (83.13) | 39 (7.93) | 44 (8.94) |
| 10. At least 1 meter/ 3 feet is the recommended social distance or physical distance for COVID-19 if go outside of home. | 420 (85.37) | 23 (4.67) | 49 (9.96) |
| 11. Individual should wash hand frequently after coming from outside, before eating or touching mouth, nose, or eyes to prevent the infection. | 450 (91.46) | 18 (3.66) | 24 (4.88) |
| 12. Recommended time for washing hand with soap/ alcohol is minimum 20-30 seconds to prevent the infection. | 388 (78.86) | 50 (10.16) | 54 (10.98) |
| 13. Isolation and supportive treatment are effective ways to reduce the spread of the virus. | 395 (80.28) | 25 (5.08) | 72 (14.63) |
| 14. The immediate observation period is 14 days if anyone contact with someone infected with the COVID-19. | 418 (84.96) | 18 (3.66) | 56 (11.38) |
| Attitudes related questions | Agree,  n (%) | Disagree,  n (%) | Don't know, n (%) |
| 1. I agree that COVID-19 will finally be successfully controlled across the world. | 337 (68.5) | 24 (4.88) | 131 (26.63) |
| 2. I have confidence that Bangladesh will win the battle against the COVID-19. | 272 (55.28) | 56 (11.38) | 164 (33.33) |
| Practices related questions | Yes,  n (%) | No,  n (%) | Sometimes,  n (%) |
| 1. When I went out, I have avoided crowded place. | 153 (42.62) | 196 (54.6) | 10 (2.79) |
| 2. When I went out, I have maintained the recommended social distance of 1 meter or 3 feet. | 229 (63.79) | 86 (23.96) | 44 (12.26) |
| 3. When I went out, I have worn a mask regularly and thoroughly. | 256 (71.31) | 47 (13.09) | 56 (15.6) |
| 4. If I were to go out, I have washed my hand after coming from outside and before eating or touching mouth, nose or eyes regularly and thoroughly. | 273 (76.04) | 4 (1.11) | 82 (22.84) |
| 5. I have maintained the recommended hand washing time of 20-30 seconds regularly and thoroughly. | 227 (63.23) | 55 (15.32) | 77 (21.45) |
